# Supplementary material for: Medical Error Disclosure: An Entrustable Professional Activity During an Objective Standardized Clinical Examination for Clerkship Students
Source: MedEdPORTAL. 2024 Feb 20;20:11382. doi: 10.15766/mep_2374-8265.11382 (PMC10876916; doi:10.15766/mep_2374-8265.11382)
Supplement: Supplementary file 1 — Faculty OSCE Guide.docxError Disclosure Standardized Patient Case.docxFaculty OSCE Checklist.docxCase-Based Experience Faculty Guide.docxCase-Based Experience Debrief Case.docxCase-Based Experience Observer Checklist.docxStudent Survey.docx [file mep_2374-8265.11382-s001.zip › B. Error Disclosure Standardized Patient Case.docx]

Appendix B: Error Disclosure Standardized Patient Case

Date: 2019

Primary Case Author: Gino Farina

Secondary Case Author: Rebecca Dougherty

Standardized Patient Educator: Terry Wanamaker

Name of Case: School of Medicine MS4 EPA

Name of Educational and/or Assessment Activity: EPA

Patient Name: Maria/Michael Miller

Chief Complaint: “My doctor sent me here for a blood transfusion”

Most Likely Diagnosis and Differential With Rationale From History and/or Physical Exam: Anemia due to upper GI bleed secondary to non-steroidal anti-inflammatory use.

Challenge Question: Challenging Questions: “Can I get HIV from the transfusion? What happens if I don’t get the blood transfusion? I appreciate that you apologized, but how did this happen?”

Domains: Check all that apply

- **Professionalism**
- **Communication and Interpersonal Skills**
- **Medical History**
- Physical Exam
- **Shared Decision-Making**
- **Patient Education**
- **Clinical Reasoning**
- **Documentation**
- **Handoff**
- **Presentation**
- Other:

Type and Level of Learner: Fourth Year Medical Student

Case Objectives: Please list specific objectives for each of the domains you have checked above:

1. Obtain informed consent from the patient

2. Place an IV and draw blood on task trainer, place blood in container for type and cross.

3. Complete an error disclosure

| SETTING: outpatient, in patient, ED, home, nursing home, rehab, group, etc. | Hospital |
| --- | --- |
| PATIENT PROFILE: Information about the “patient” that helps select an SP and helps the learner get an understanding of them as a person. SP will know more information about the patient than learner will ever ask but allows SP to portray a fully developed patient personality. If none of the items below are particulars for the case, please write “all may be used.” | |
| Age range | Late 40’s, early 50’s |
| Religious/spiritual background | N/A |
| Sex (e.g., male, female, intersex, transwoman, transman) | Male, Female |
| Sexual orientation (e.g., heterosexual, lesbian, gay, bisexual, pansexual, queer, asexual) | Heterosexual |
| Gender expression (e.g., man, woman, genderqueer) | Man or Woman |
| Race and ethnicity | N/A |
| Physical description (e.g., BMI, height range) | N/A |
| Physical limitations | None |
| Patient appearance (e.g., disheveled, hospital gown, business casual, casual) | Hospital gown |
| Moulage + location (e.g., none, bruises, scars, body piercing, tattoos) | None |
| Affect (e.g., pleasant, cooperative) | Cooperative |
| Family group (e.g., who is family, who they live with) | Married to spouse for 15 years. Has three children, all boys, ages 8, 10, 12. |
| Education | College graduate |
| Level of health literacy | High |
| Employment, if any - present and past, noting any current stresses | Certified Public Accountant |
| Home/homeless - type of dwelling, number of stories, owned or rented | Owns ranch-style home. |
| Financial situation - any current stresses | No financial stress |
| Insurance status (e.g., un/under/insured, public/private, HMO/PPO) | Insured |
| Habits (i.e., diet, exercise, caffeine, smoking, alcohol, drugs) | Occasional wine, 1 glass per week. No tobacco or drug use. Minimal caffeine use. Exercises regularly. |
| Activities (i.e., hobbies, sports, clubs, friends) | Spending time with children, enjoys attending professional hockey games |
| Typical day - what is the usual daily routine | Work, exercise, time with children. |

| CASE INFORMATION | |
| --- | --- |
| Chief Concern: What the patient will say when greeted by the student. The patient’s primary reason for seeking medical care often stated in their own words. | “My doctor sent me here for a blood transfusion.”  I had a follow up appointment with my doctor for a knee injury and he did some blood work because he said I looked a bit pale. He called me today and said that I should come to the ED for a blood transfusion. |
| Additional Concerns: Other, if any, concerns the patient has today (i.e., symptoms, requests, expectations, etc.) that will become part of set agenda. | None. |
| THE PATIENT’S STORY: The SP will be asked to tell their symptom story and the personal and emotion impact for each of their concerns. You will want to write this in the patient’s voice. The symptom story should be able to answer this question: “Tell me more about [chief concern/additional concern], starting at the beginning and bringing me up to now.”  The personal context should be able to answer questions concerning the broader personal/psychosocial context of symptoms, especially the patient’s beliefs/attributions.  The emotional context should be able to ask how are you doing with this, how does this make you feel, how has this affected you emotionally? IMPACT: How has this affected your life? How has this been for your family? | Please note: The purpose of the exercise is for students to 1) obtain informed consent to perform a blood transfusion, 2) place an IV/draw blood on the task trainer 3) notice an error in the blood labels and 4) inform you that an error was made in the blood labeling.  You have been well until around 5 weeks ago when you twisted your right knee playing basketball with your son. The knee swelled up right away so you went to your doctor who performed an x-ray (that you were told was negative). The doctor gave you ibuprofen. You have been taking 600 mg every 8 hours around the clock with gradual relief of your symptoms. Pain was 8/10 and sharp. Now it’s a 2/10 and dull.  Approximately 3 weeks ago you began to notice some pain in your stomach area (5/10 and achy) about 1-2 hours after eating. It appeared to get worse if you also had wine with the meal.  1 week ago you noticed that your stools were getting really black – almost looked like “tar.” You have attributed this to eating more spinach and vegetables lately.  For the last few days you have been feeling a bit lightheaded when you stand up from a sitting or lying down position. This quickly resolves after 15 seconds. You have not passed out. You are not nauseous and have not vomited. You have no chest pain. You haven’t really exercised lately because of your knee, but you notice that you get short of breath with some exertion – for example one of the elevators was out at the office building you work at and you had to take the stairs up 2 flights – you were very short of breath and had to stop once going up midway.  Your knee is much better. The swelling resolved after about 1 week on the ibuprofen. Your knee pain is almost completely gone. You notice it only after walking a lot – for example you were walking around the city over the weekend as you and your spouse had tickets to a concert for your anniversary and by the time you came home you needed to take more ibuprofen.  (You also felt a bit short of breath when you exerted yourself too much – when you were walking too quickly.)  Currently you feel fine and you are not dizzy sitting in the room.  Since being here today, you first IV infiltrated and the student coming in has to give you a new IV and draw your blood. |
| HISTORY OF PRESENT ILLNESS: Although some of the HPI will be given in the patient’s symptom story, the learners will expand the story during the direct question section. Below, describe the detailed history, usually about the chief concern, which the student must develop in order to make a useful assessment of the problem: | |
| Onset (when; gradual or sudden) | 5 weeks ago, gradual |
| Setting (what was going on or where was patient when symptoms first noticed?) | Approximately 3 weeks ago you began to notice some pain in your stomach area (5/10 and achy) about 1-2 hours after eating. It appeared to get worse if you also had wine with the meal. |
| Duration (how long) | 5 weeks |
| Time relationships (frequency, constant or intermittent) | For the last few days you have been feeling a bit lightheaded when you stand up from a sitting or lying down position. This quickly resolves after 15 seconds. You have not passed out. You are not nauseous and have not vomited. You have no chest pain. You haven’t really exercised lately because of your knee, but you notice that you get short of breath with some exertion – for example one of the elevators was out at the office building you work at and you had to take the stairs up 2 flights – you were very short of breath and had to stop once going up midway. |
| Location | Knee pain |
| Radiation | None |
| Quality | Your knee is much better. The swelling resolved after about 1 week on the ibuprofen. Your knee pain is almost completely gone. You notice it only after walking a lot – for example you were walking around the city over the weekend as you and your spouse had tickets to a concert for your anniversary and by the time you came home you needed to take more ibuprofen.  (You also felt a bit short of breath when you exerted yourself too much – when you were walking too quickly.)  Currently you feel fine and you are not dizzy sitting in the room. |
| Amount | N/A |
| Aggravated by what | Position |
| Relieved by what | Lightheadedness, dizziness, shortness of breath decreased by decreasing activity |
| Associated with what | Lightheadedness, dizziness, shortness of breath |
| Attitude (what does the patient think is the problem, and how do they feel about it) | You had thought you were tired and short of breath because you were out of shape because of the knee injury |
| Overall course | Sent to ED by doctor for blood transfusion |
| REVIEW OF SYSTEMS: Significant positives and negatives | |
|  | All systems other than those covered in HPI are negative |
|  |  |
| Past medical history | None |
| Medication allergies (name and reaction) | No allergies to medications |
| Environmental allergies (name and reaction) | Allergies to pollen |
| Illnesses | None |
| Vaccinations | You had all of your childhood vaccinations. Your last flu vaccine was last year. You have never had the vaccine for pneumonia. You received both doses of your COVID-19 vaccine (Moderna) June 2021 and receive booster(s) as needed. |
| Surgeries | None |
| Accidents/injuries/trauma | None |
| Hospitalization | None |
|  | |
| Inclusive sexual and reproductive history | |
| Sexual practices  Sexual partners  Protection: Use of safer sex practices  Use of birth control if appropriate  Risk of intimate partner violence | Vaginal  3 lifetime partners  None  Spouse with vasectomy  None |
| OB/GYN history | Age of onset of menses  Age of menopause  Number of pregnancies  Number of live births  Number of miscarriages  Number of abortions |
| Medications | Prescription/dose/reason: None  Over the counter/dose/reason: Ibuprofen 600 mg (3 pills) up to every eight hours, now only occasionally for right-sided knee pain as described in HPI  Herbs/supplements/dose/reason: None  Other: |
| Immunizations | - **Tetanus** - **Flu** - **Hepatitis** - Pneumovax - HPV - Other: You had all of your childhood vaccinations. Your last flu vaccine was last year. You have never had the vaccine for pneumonia. You received both doses of your COVID-19 vaccine (Moderna) June 2021 and receive booster(s) as needed. |
| Tobacco products:   - Cigarettes - Cigar - Pipe - Chew - E-cigarettes | - **Never** - Past - year started/year quit - Current   - Quantity   - # of years |
| Alcohol   - Beer - **Wine** - Liquor - Other | - Never - Past - year started/year quit - Current   - Quantity: 1 glass a week   - # of years: 20 years |
| Drugs   - Weed - Cocaine - Heroin - Meth - IV - Inhalants - Other | - **Never** - Past - year started/year quit - Current   - Quantity   - # of years |
| Diet (describe) | Diet is fair, try to eat a variety of foods – doesn’t make limits. Lately has gotten onto a health kick. Started making green shakes out of vegetables – and added a lot of vegetables and fruits into diet. Felt since the injury that this was a realization that you were getting older and need to be more mindful of your health |
| Exercise (describe) | 30 minutes of aerobic exercise four times a week |
| List any other important social history or information important to this case | None |
| Family history | Father, 65 and Mother, 68 both alive and well with high blood pressure and high cholesterol.  No siblings  3 children – all boys – alive and well. Michael, John, Peter 8,10,12 |
| Mother, father, siblings, grandparents, and other significant findings | Father, 65 and Mother, 68 both alive and well with high blood pressure and high cholesterol.  No siblings  3 children – all boys – alive and well. Michael, John, Peter 8,10,12 |
|  |  |
| Physical Exam - List exam maneuvers expected for this case and any abnormal findings that SP will simulate. (tenderness, hyper-hypo reflex, rebound, weakness, etc.)  None | |
| PHYSICAL EXAM FINDINGS |  |
| 1. Written in layperson’s terms | None |
| 1. General appearance - affect, appearance, position of patient at opening (i.e., sitting, lying down, holding abdomen, etc.) | Pleasant, not in distress, siting on hospital table |
| 1. Vital signs | None |
| 1. Specific findings and affect | None |
| 1. Response to certain physical movements | None |
|  |  |
| DIAGNOSIS AND DIFFERENTIAL |  |
| Diagnosis with support from positive and negative history and PE findings | N/A |
| Differential with support from positive and negative history and PE findings | N/A |
|  |  |
| MANAGEMENT OR DIAGNOSTIC PLAN | 1) obtain informed consent to perform a blood transfusion, 2) place an IV/draw blood on the task trainer 3) notice an error in the blood labels and 4) inform you that an error was made in the blood labeling. |
|  |  |
| PROFESSIONALISM ISSUES OR CHALLENGES | Error disclosure, see below |

Additional Case notes

| Encounter timing: | 25 min (both parts take place in this 25 min time frame): 1) informed consent & blood draw 2) error disclosure – 2 min warning  Learner & Faculty are both in the room for the encounter and briefly leave the room between these two sections |
| --- | --- |
| Post-encounter timing: | N/A (no checklist) |
| Feedback details: | 15 min Feedback w/ student, SP, & faculty – 2 min warning |

| Opening Statement/ Chief Concern | “My doctor sent me here for a blood transfusion” |
| --- | --- |
| Impact/Concern/ Explanatory Model | Impact: Now that you mention it – I realize that I was a bit short of breath walking around the city – and I really took it down a notch and walked much more slowly.  Concerns: You are concerned about the need for a blood transfusion.  Explanatory model: You had thought you were tired and short of breath because you were out of shape because of the knee injury |
| SP instructions: | SP will have red gown over their clothes. (If working at the School of Medicine, you can use your regular gown) Every SP should have a pen in the room.  Task trainer will be in the room and all materials laid out. The ZSOM coordinators will reset the materials between each encounter. (Please note: feedback will take place across the hall in a different exam room so the coordinators have time to re-set the arms)  Nurse (ZSOM Faculty) will be in the room with you  If the student tries to perform a physical exam, please say: *“*I’m not here for that today.” Do not allow the student to perform any physical exam items. |
| Informed Consent: | In the first portion of the encounter the learner will obtain your informed consent for a blood transfusion. To do this they will explain why this procedure is necessary and discuss the risks, benefits, and alternatives to the procedure. They may start the encounter by asking an opened ended question such as “what do you know about why you need a blood transfusion?”.  If asked what you know about why you need the blood transfusion: You know you’ve been feeling a bit off and looked pale so your doctor ran some blood work which determined that you need a blood transfusion.  You are not averse to any of the procedures. After the student has explained the risks, benefits, and alternatives, they may ask if you have any questions. If the student doesn’t spontaneously explain the following information, at some point during the consent process you should ask:  Challenging Questions:  1. “Can I get HIV from the transfusion?” (risks)  2. “What happens if I don’t get the blood transfusion?” (alternatives)  Teachback: Learner may ask you to repeat back what you have discussed to confirm your understanding.  After addressing your questions and doing teachback the student will ask for your verbal and written consent. Please make sure you bring a pen with you into the room to sign the student’s form. |
| Error Disclosure: | After the student obtains your informed consent and draws blood from the task trainer, the faculty will tell them there was an error in the way the blood is labeled and they must go back in the room to tell the patient about the error.  Please note: these students are 4^th^ year medical students and may not have reviewed this curriculum for some time. You may have some students who are very comfortable with how to disclose an error and some who are not. Please do not continue to escalate your frustration too much if you have a learner who is not well versed in how to perform this task. You should remain at about 3-4 out of 10 on the scale of anger throughout the error disclosure. If your learner completes the steps below this will slowly deescalate your anger.  If the student asks: What is your understanding of what happened to the blood work?  You respond: “All I know is that you did the test and said you were bringing those to the lab right away and now you have to re-do the test. Does anyone here know what they are doing?” (Note to SP: the Nurse told you a new blood draw is needed and you have been waiting for the student come back in and explain why.)  If the student asks: May I summarize the events up to this point?”  You respond: “Sure, please do.”  The student will share the medical error, ideally clearly and concisely.  If the student is not clear and to the point, or tries to sugar coat the medical error as if it is not a big deal, say: "Wait, wait –what are you trying to say?”  ****ONCE THE STUDENT SHARES THE ERROR— shake your head and allow for a pause before you respond  If the student pauses for your response:  You will respond: “I am so upset that this happened. This whole visit has been very frustrating and stressful.”  If the student does not pause for your response: refrain from verbally sharing your emotional response, but express that you are displeased by rolling your eyes and shaking your head, state “I just can’t believe this happened.”  The students are expected to utilize the skills of empathy in response to the emotion just expressed, following the pause. If the student utilizes the skills of empathy (i.e. “I can see that you are frustrated, I would be frustrated as well, what is frustrating you the most,” you will respond:  The delay in care is very frustrating.  The student must 1) apologize for the medical error and 2) acknowledge responsibility for the error to complete their task correctly. An apology alone is not the same as acknowledging responsibility, and acknowledging responsibility is not the same as saying they are sorry.  The student can either acknowledge responsibility by either stating that the team did not ensure that the specimen was labelled correctly or may take personal responsibility stating:  “I forgot to label the specimen; It’s my fault, I acknowledge that I did not handle the blood sample properly.”)-- the critical piece here is an acknowledgement that an error occurred.:  You respond: I am upset that the sample was not properly handled.  ***Challenge Question*** “I appreciate that you apologized, but how did this happen?  At some point during the encounter you can also add: “This whole experience has been awful, and I am very frustrated by the care I have received.” (Appear frustrated)    If the student provides an explanation as to why the error occurred (i.e. May I share what is known about why the specimen was not labelled correctly?”)  You will listen to the student’s explanation and respond “It sounds like a lot of steps and chances for something to go wrong.”  If the student describes what will be done to immediately address the error (i.e. “May I share what I plan to do to immediately address what happened? I am going to re-draw the blood and label the specimen immediately after checking that your name and date of birth match the label.”):  You respond: “Thank you for your honesty and the immediate actions that you are going to take.”  If the student discusses the need to perform a repeat blood draw (I.e. I am so sorry, but I need to repeat the blood draw.)  You respond: “Yes, I guessed that would have to happen. It needs to be done.”  If the student outlines the steps that will be taken to prevent recurrence, (i.e., “May I share what I plan to do to prevent this from happening again? I plan to label all specimens in immediately after obtaining them after checking to make sure the name and date of birth on the label match the patient’s.”):  You say, “I guess, I don’t want this to happen to another patient.”  After they share say, “Thank you for sharing, I’m glad you have a plan on how to prevent this from happening again.  Not relevant in this case but if the student offers support services (i.e. “Would you like me to connect you with some of our support services like our office administrator”):  You respond: “Thank you, but I think I am fine for now. |
| Prop instructions | Wrist band on patient  NAME: MICHAEL / MARIA MILLER  DOB: 2/21/1975  Wrong Labels  NAME: MITCHELL/ MARSHA MILLER  DOB: different DOB   - - - Task trainer for IV and blood draw     - Wrist band correctly labeled     - Two sets of labels – one correctly labeled and one incorrectly labeled     - Tubes for T&C     - Consent form for transfusion     - IV equipment     - Extra blood tubes   Trainer Note: PSI supplies the task trainer and blood draw supplies. CSC should email PSI to confirm task trainer delivery one week prior to the event. The ZSOM provides wrist bands, labels, consent forms and sharps containers. CSC books/trains SPs and supplies red robes and pen. Events occur at CSC or ZSOM. |
| Faculty Handoff (There is no door chart for this event) | The student is told by the faculty member during sign out to obtain informed consent for the blood transfusion (student will be given blank consent form), draw a Type and Cross on the task trainer, and replace the patient’s IV which has infiltrated. (Note to SP: we used to use gauze to simulate the infiltrated IV. We no longer do this, but you should still know this background information for your encounter.)  The faculty member will be in the room in the role of the nurse to hand the learner the tubes and labels for the blood draw and tell the student that the IV infiltrated.  The following medical error has been built into the case: The patient’s correct date of birth as given orally by the SP and written on the SP’s wrist band differs from that on the labels for the Type and Cross.  In the rare event that the student notices the error on the blood label during the first part of the encounter, the faculty will ask the student to pretend they did not find the error so they can still practice disclosing the error to the patient. |
