# Supplementary material for: Medical Error Disclosure: An Entrustable Professional Activity During an Objective Standardized Clinical Examination for Clerkship Students
Source: MedEdPORTAL. 2024 Feb 20;20:11382. doi: 10.15766/mep_2374-8265.11382 (PMC10876916; doi:10.15766/mep_2374-8265.11382)
Supplement: Supplementary file 1 — Faculty OSCE Guide.docxError Disclosure Standardized Patient Case.docxFaculty OSCE Checklist.docxCase-Based Experience Faculty Guide.docxCase-Based Experience Debrief Case.docxCase-Based Experience Observer Checklist.docxStudent Survey.docx [file mep_2374-8265.11382-s001.zip › C. Faculty OSCE Checklist.docx]

Appendix C: Faculty OSCE Checklist

| **EPA 13-Identify System Failures and contributes to a culture of safety and improvement**  Did the student use 2 patient identifier when labelling the blood tube?  No  Yes  Did the student recognize the error?  No  Yes  **Disclosing an error**  Did the student explore the patient’s understanding of what happened?  No  Yes  Did the student disclose the error using clear language?  No  Yes  Did the student discuss steps being taken to manage the event?  No  Yes  Did the student express regret/offered an apology?  No  Yes  Did the student discuss future steps to avoid a similar event?  No  Yes  Did the student utilize the micro-skills of empathy during the encounter?  No  Yes  **Please rate the student’s ability to disclose an error using the following entrustability scale:**  Can only observe someone doing it  Can do it if supervisor talks them through it  Can do it with supervisor is in room to direct them from time to time  Can do it with supervisor available just in case  Can do it without supervisor in the room |
| --- |
