# Supplementary material for: Medical Error Disclosure: An Entrustable Professional Activity During an Objective Standardized Clinical Examination for Clerkship Students
Source: MedEdPORTAL. 2024 Feb 20;20:11382. doi: 10.15766/mep_2374-8265.11382 (PMC10876916; doi:10.15766/mep_2374-8265.11382)
Supplement: Supplementary file 1 — Faculty OSCE Guide.docxError Disclosure Standardized Patient Case.docxFaculty OSCE Checklist.docxCase-Based Experience Faculty Guide.docxCase-Based Experience Debrief Case.docxCase-Based Experience Observer Checklist.docxStudent Survey.docx [file mep_2374-8265.11382-s001.zip › E. Case-Based Experience Debrief Case.docx]

Appendix E: Case-based experience debrief case

You performed a Lumbar Puncture (LP) on an 18-year-old college student who is accompanied by his mom to rule out meningitis. The LP tubes were labeled with the incorrect patient information. As a result, the tubes were received under the wrong patient’s information. This error was not immediately recognized. You must inform the Parent/Patient and repeat the LP.
