# Supplementary material for: Medical Error Disclosure: An Entrustable Professional Activity During an Objective Standardized Clinical Examination for Clerkship Students
Source: MedEdPORTAL. 2024 Feb 20;20:11382. doi: 10.15766/mep_2374-8265.11382 (PMC10876916; doi:10.15766/mep_2374-8265.11382)
Supplement: Supplementary file 1 — Faculty OSCE Guide.docxError Disclosure Standardized Patient Case.docxFaculty OSCE Checklist.docxCase-Based Experience Faculty Guide.docxCase-Based Experience Debrief Case.docxCase-Based Experience Observer Checklist.docxStudent Survey.docx [file mep_2374-8265.11382-s001.zip › F. Case-Based Experience Observer Checklist.docx]

Appendix F: Case-Based Experience Debrief Observer Checklist

| Components of Error Disclosure | Y/N | Comments: |
| --- | --- | --- |
| Explored patient’s understanding of what happened. |  |  |
| Disclosed the error using clear language. |  |  |
| Discussed steps being taken to manage the event. |  |  |
| Expressed regret/offered an apology. |  |  |
| Discussed future steps to avoid a similar event. |  |  |
| Utilized the micro-skills of empathy during the encounter. |  |  |
