# Supplementary material for: Medical Error Disclosure: An Entrustable Professional Activity During an Objective Standardized Clinical Examination for Clerkship Students
Source: MedEdPORTAL. 2024 Feb 20;20:11382. doi: 10.15766/mep_2374-8265.11382 (PMC10876916; doi:10.15766/mep_2374-8265.11382)
Supplement: Supplementary file 1 — Faculty OSCE Guide.docxError Disclosure Standardized Patient Case.docxFaculty OSCE Checklist.docxCase-Based Experience Faculty Guide.docxCase-Based Experience Debrief Case.docxCase-Based Experience Observer Checklist.docxStudent Survey.docx [file mep_2374-8265.11382-s001.zip › G. Student Survey.docx]

Appendix G: Student Survey

|  | Error Disclosure Survey | Strongly Disagree | Disagree | Neither Agree nor Disagree | Agree | Strongly Agree |
| --- | --- | --- | --- | --- | --- | --- |
| 1 | It is essential for health care providers to know how to disclose an error. |  |  |  |  |  |
| 2 | I know how to disclose an error to a patient and/or a patient’s family. |  |  |  |  |  |
| 3 | Health care providers should apologize to patients following an error. |  |  |  |  |  |
| 4 | I feel comfortable disclosing an error to a patient and/or family member |  |  |  |  |  |

|  |  | Yes | No |
| --- | --- | --- | --- |
| 5 | I feel confident that I can identify an error |  |  |

Free text response:

| 6 | If yes, what did you do and why? Did you feel comfortable speaking up? |
| --- | --- |
| 7 | If no, why do you think you didn’t identify it? How did you feel |
| 8 | Please reflect on the experience of disclosing the error. How did you feel? |
