# Supplementary material for: Medical Error Disclosure: An Entrustable Professional Activity During an Objective Standardized Clinical Examination for Clerkship Students
Source: MedEdPORTAL. 2024 Feb 20;20:11382. doi: 10.15766/mep_2374-8265.11382 (PMC10876916; doi:10.15766/mep_2374-8265.11382)
Supplement: Supplementary file 1 — Faculty OSCE Guide.docxError Disclosure Standardized Patient Case.docxFaculty OSCE Checklist.docxCase-Based Experience Faculty Guide.docxCase-Based Experience Debrief Case.docxCase-Based Experience Observer Checklist.docxStudent Survey.docx [file mep_2374-8265.11382-s001.zip › A. Faculty OSCE Guide.docx]

Appendix A: Faculty OSCE Guide

Faculty Guide: EPA Exercise

Preparation for Residency

Table of Contents:

Clinical Skills Exercise Guide

Learning Objectives and Overview

I-PASS Handoff for Faculty

Faculty Debrief Guide for Clinical Skills Exercise

Overview of the EPA Exam

*Clinical Skills Exercise*

In the clinical skills center, the faculty observes as the student encounters a standardized patient in need of a blood transfusion and hospital admission for severe symptomatic anemia.

The exercise begins with the student receiving a verbal patient handoff from the faculty member. The student must then enter the patient’s room and obtain informed consent from the patient for a blood transfusion (*EPA 11*). The patient requires a blood draw and IV placement (*EPA 12*) which the student will perform on a task trainer (mannequin). A medical error is built into the encounter. The student will disclose the medical error to the patient. (*EPA 13*). The exercise concludes with the faculty member facilitating an individual debrief of the exercise focusing on the tested EPA skills.

At the completion of the exercise, the student is expected to write admission orders for the anemia case (*EPA 4*). The student will have the opportunity to compare his or her orders with suggested versions.

Detailed Agenda

*Clinical Skills Exercise*

0-20 Minutes: The Student receives a verbal I-PASS handoff from the Faculty Member on the standardized patient in need of a blood transfusion and hospital admission (written version of the handoff will be provided to faculty). The student enters a room and encounters the standardized patient. The Faculty Member enters the room with the student and acts as the nurse, coaching the student on placing an IV and drawing blood on a task trainer as needed. The student exits the room with the nurse. The scenario continues several hours later where the nurse informs the student that the blood draw was mislabeled** and instructs the student to share this information with the patient. The nurse then demonstrates proper procedure for blood draws. (***Note that this scenario continuation should occur even if the student notes the incorrect label.)*

*Clinical Skills Debrief*

20-35 Minutes: The Student and Faculty Member enter the debrief room and the Faculty Member debriefs the Student on the Clinical Skills exercise focusing on the tested EPA skills (detailed Debrief Guide below).

Clinical Skills Exercise Guide

Learning Objectives:

At the completion of this session, learners will meet or identify gaps in their ability to perform the following objectives:

1. Enter and discuss patient orders (EPA 4)
2. Obtain informed consent for tests and/or procedures that the day 1 intern is expected to perform or order without supervision (EPA 11)
3. Perform general procedures of a physician (EPA 12)
4. Identify system failures and contribute to a culture of safety and improvement (EPA 13)
5. Disclose a medical error to a patient (EPA 13)

Overview of Standardized Patient Encounter (total of 35 minutes including verbal I-PASS handoff from Faculty Member):

The scenario has two parts *(see detailed summary below).* The first part includes providing a verbal handoff in I-PASS format, the student interaction with the SP discussing informed consent for a blood transfusion and performing a blood draw on a mannequin arm. The second part includes the student completing an error disclosure.

PART 1: 10 minutes

After receiving a verbal handoff in the I-PASS format from the Faculty Member (provided below), the student will see Michael or Maria Miller, a man or woman around 50 years of age with symptomatic severe anemia secondary to a GI bleed due to NSAID use.

After providing the student with the verbal I-PASS handoff, the faculty member will then assume the role of the *nurse* in the standardized patient encounter and thus be physically present in the room with the student during the encounter. The purpose of the faculty member playing the nurse is to provide the student with assistance and guidance with the blood draw and IV placement if needed.

Students will be given the following background from the Faculty Member prior to seeing the patient (the objective of the exercise is NOT for the student to obtain a history and physical so this information will be provided):

The patient has been sent to the ED by his/her primary care doctor for a blood transfusion and hospital admission secondary to a Hb of 5.3 g/dL with dyspnea on exertion and weakness. The underlying etiology is thought to be a GI bleed secondary to NSAID overuse for a knee injury. The patient is orthostatic, tachycardic, and guaiac positive. EKG was normal.

The student is told by the Faculty Member during sign out to obtain informed consent for the blood transfusion (student will be given blank consent form), draw a Type and Cross on the task trainer, and replace the patient’s IV which has infiltrated.

The Faculty Member will be in the room in the role of the nurse to hand the patient the tubes and labels for the blood draw and tell the student that the IV infiltrated.

The following medical error has been built into the case: The patient’s correct date of birth/MRN as given orally by the SP and written on the SP’s wrist band differs from that on the labels for the Type and Cross. The faculty member as nurse will thus hand the student an incorrect label. The student should identify the error by cross checking the label with the patient’s wrist band prior to drawing the blood. If the student does not identify the error, the faculty member playing the patient/nurse will not point it out. If the student does identify the error, the faculty member playing the nurse will acknowledge that the student is correct, briefly leave the room, and return with new correct labels. Faculty Role during the Clinical Skills Encounter:

1. Faculty should act as the nurse in the room.
   1. Faculty should sign the informed consent as witnessed if asked to by the student.
   2. Faculty should hand the student the empty blood draw tubes and incorrect labels. If the student identifies the error, faculty should acknowledge the mistake, briefly leave the room, obtain the correct labels right outside the door, and return to the room and hand the student the correct labels.
   3. Faculty should act in the capacity of a nurse-if the student is having trouble with the blood draw and IV placement, provide guidance on correct procedure. DO NOT perform the procedure for the student.
2. While in the room, faculty should observe each student’s encounter noting areas of strength and need for improvement. Please pay particular attention to whether the student discussed all aspects of informed consent, whether the student noticed and responded to the medical error, and the skill level of the blood draw and IV placement.

PART 2: 10 minutes

Upon conclusion of the initial encounter, the faculty and student will exit the room. Outside the room, the faculty (still playing the role as nurse) will share the following with the student:

- “The blood was drawn and labeled with the incorrect patient information. As a result, the type and cross was received under the wrong patient’s information. This error was not recognized for several hours. As this patient’s nurse, I let the patient know that another blood draw is needed. Can you please share this with the patient? Once you let the patient know I will re-draw blood from the IV.” (after student discloses the error, acting as the nurse demonstrate the procedure is done correctly.) ****This information should be shared with the student even if the student identified the error. The student’s recognition of the incorrect label should be acknowledged and commended, however the goal of the next portion of the encounter is to have the student disclose this error to the patient. The student will therefore be asked to imagine that s/he did not notice the incorrect label.*

The faculty (acting as the patient’s nurse) will then instruct the student to re-enter the room with the SP and disclose the error to the patient. The faculty (acting as the patient’s nurse) will accompany the student into the room.

Upon the student’s completion of the error disclosure, the Faculty (acting as the patient’s nurse) will demonstrate the proper procedure of verifying the patient’s identity with the labels.

Debrief: 15 minutes

The exercise will conclude with a 15 minute faculty facilitated debrief of the Student focusing on the tested EPA skills. The debrief will occur in the patient room and the SP will offer the patient’s perspective on the encounter. Detailed Debrief Guide follows.

I-PASS Handoff for EPA Anemia Clinical Skills Case

Note that the Faculty Member will provide the following handoff to all each Student at the start of the Clinical Skills exercise.

The Faculty Member should begin by telling the Student that you are in the ED and that you (the Faculty Member) are the ED resident taking care of this patient. You are going off shift and thus signing the patient out to the student who is the intern coming on shift.

Instruct the student to take notes and that the handoff format is I-PASS.

Patient: MARIA MILLER or MICHAEL MILLER. DOB: 2/21/75 MR: 5624125

Allergies: None

Illness severity

This patient is a “watcher,” please keep your eye on her/him.

Patient summary

Mr/Ms. Miller is a man or woman with a history of a knee injury who was sent to the ED by his/her PCP for evaluation for a blood transfusion.  He/she was taking NSAIDs for a while due to the knee injury and presented to his/her PCP today complaining of dyspnea on exertion and weakness. The PCP ordered labs and he/she was found to have a Hgb of 5.3 so the PCP sent him/her in to the ED. He/she probably has a GI bleed from the NSAID use.  She/He is orthostatic, tachycardic and guaiac positive.  His/her EKG did not reveal ischemia and she/he is being admitted for a blood transfusion and GI work up. He/she is stable but requires admission for the transfusion and monitoring.

Action list

Anemia is the only active issue. She needs to be transfused 2 units of PRBCs and GI will need to see her for endoscopy.

Situation awareness and contingency Plans

If she becomes hemodynamically unstable she will need the transfusion stat and we will have to transfer her to the MICU.

At this point we are giving her fluids, have ordered a blood transfusion, and a GI consult.  I need you to obtain the informed consent for the blood transfusion.  I also need you to draw the Type & Cross. The first tube was sent from the ED but she needs a second confirmatory tube sent to the blood bank before they release the blood. Also, her IV fell out so please replace.

I think the nurse is in there with her now making sure we have everything set up.  Here is a copy of the consent form. *(Hand the student the form.)*

Synthesis by receiver

This is an opportunity for the Student to ask the Faculty Member questions. Faculty should ask, “Do you have any questions?”

Students should summarize/read back what they just heard. If they do not, please prompt by saying, “Can you please read back to me what you just heard so I can make sure we are on the same page?”

*At this point, please step out of character and inform the student that you will be assuming the role of the nurse in the standardized encounter.*

Faculty Debrief Guide for Clinical Skills Exercise

Timing: 15 minutes, please note that the following is a suggested guide for debrief but time may not allow all topics to be covered.

Format:

The debrief should focus on *process* (the EPAs) not *medical content.* If students gear the conversation toward medical content (ie: patient management), please redirect them by saying, “That is an excellent question for you to read about. For today, we will be focusing on the EPA skills, not medical content.”

1. Frame the debrief around the EPAs covered in this session:

“This exercise covered the following EPAs:

Obtain informed consent for tests and/or procedures that the day 1 intern is expected to perform or order without supervision (EPA 11)

Identify system failures and contribute to a culture of safety and improvement (EPA 13)

Disclose a medical error to a patient (EPA 13)

Perform general procedures of a physician (EPA 12)

Enter and discuss patient orders (EPA 4).”

1. Continue with a discussion of the informed consent process.
   1. “How did the process of obtaining informed consent go for you?”
   2. “What constitutes informed consent?” Attempt to elicit the following components and ask the students to reflect upon whether they completed all of them
      1. Indications for the procedure: *anemia*
      2. Risks of the procedure: *transfusion reaction, low risk of infection (Hepatitis, HIV at minimum; CMV/EBV if fancy)*
      3. Benefits of the procedure: *symptomatic relief and prevention of cardiac complications of severe anemia*
      4. Alternatives to the procedure: *nothing, more saline, or iron tablets which would not help in short term*
      5. Ensuring patient understanding of above: *teach back*
      6. Obtaining patient and witness signatures
2. Continue with a discussion of the medical error.
   1. “How did the process of disclosing an error to the patient go for you?”
   2. “What elements are essential in an error disclosure?” Attempt to elicit the following components and ask the students to reflect on whether they completed all of them.
      1. Explored patient’s understanding of what happened
      2. Disclosed the error using clear language
      3. Discussed steps being taken to manage the event
      4. Expressed regret/offered an apology
      5. Discussed future steps to avoid a similar event
      6. Utilized micro-skills of empathy during the encounter
   3. Observe if the student demonstrated the following: avoided discussing the error, defensiveness, placed blame on another individual (e.g. the nurse). If so, elicit the student’s reflection.
   4. Explore the following with the student, “What factors contributed to the error?”
   5. Explore whether or not the student knows how to seek help following an error. Share that that they should notify their senior resident and/or attending. Remind the student that there are anonymous reporting structures in place. At Northwell this is Improve North.
3. Invite the SP to offer his/her perspective on the error disclosure.
4. Continue by asking the students how the blood draw/IV placement went for them.
   1. Review the need for universal precautions (gloving, disposing of sharps appropriately).
   2. Tell the student that if he/she did not successfully draw blood or place an IV there will be an additional opportunity to practice with coaching immediately after the debrief.

References:

1. Amiel JM, Andriole DA, Biskobing DM, Brown DR, Cutrer WB, Emery MT, Mejicano GC, Ryan MS, Swails JL, Wagner DP; Association of American Medical Colleges Core EPAs for Entering Residency Pilot Team. Revisiting the Core Entrustable Professional Activities for Entering Residency. *Acad Med.* 2021 Jul 1;96(7S):S14-S21. doi: 10.1097/ACM.0000000000004088. PMID: 34183597.
