# Supplementary material for: Medical Error Disclosure: An Entrustable Professional Activity During an Objective Standardized Clinical Examination for Clerkship Students
Source: MedEdPORTAL. 2024 Feb 20;20:11382. doi: 10.15766/mep_2374-8265.11382 (PMC10876916; doi:10.15766/mep_2374-8265.11382)
Supplement: Supplementary file 1 — Faculty OSCE Guide.docxError Disclosure Standardized Patient Case.docxFaculty OSCE Checklist.docxCase-Based Experience Faculty Guide.docxCase-Based Experience Debrief Case.docxCase-Based Experience Observer Checklist.docxStudent Survey.docx [file mep_2374-8265.11382-s001.zip › D. Case-Based Experience Faculty Guide.docx]

**Appendix D: Case-based Experience, Faculty guide**

**Introductions & Reflections:**  **10 minutes**

Faculty: Ask the students to share responses to the following questions:

- What have you seen thus far in terms of error disclosure? What was done well? What wasn’t done well?
- How did you feel following the experience? What did you do to care for yourself, your team, your patient?
- How did the Clinical Skills session go?

**Discussion**  **10 minutes**

Faculty: Ask the students to share responses to the following questions:

- **What factors contribute to errors occurring? Why might this be the case? The following are examples:**
  - Provider cognitive error
  - Communication
  - Documentation
  - Patient Education/ literacy
  - Workload, staffing, EMR
- **Are there times which are particularly vulnerable to Error? Why might this be the case? The following are some examples:**
  - Transitions
  - Provider Bias
  - Physician Wellness
- **Why is disclosing an error important? The following are some examples:**
  - Maintains trust, facilitates resilience and recovery
- **What barriers are there? The following are some examples:**
  - Lack of education, discomfort of provider, embarrassment, shame, emotional distress, lack of formalized approach, lack of institutional support
  - Fear of litigation
- **What is disclosure? The following are some examples:**
  - Communication based on trust
  - Collaborative communication between provider and patient versus deny and defend
- **What are the essential components of a disclosure? The following are some examples based on AHRQ’s CANDOR toolkit^2^**
  - State in concise, patient centered language, what happened
  - Share what is known about why it happened
  - Apologize and acknowledge responsibility
  - Share what will be done to immediately address the error
  - Share what will be done to prevent recurrences
  - Utilize empathy
- **What are other important elements to consider? The following are some examples:**
  - Avoid using medical jargon, utilize patient centered language
  - Pay attention to non-verbal behaviors: Positioning, Eye contact
  - Minimize distractions: Phone/Pager, Alarms

**Re: Re-Practice in Pairs**   **10 minutes**

Invite the students to re-practice Error Disclosure in pairs utilizing case *(Appendix E)* and checklist *(Appendix F).* Ask students to take turns being the physician and the parent. Have the students evaluate their performance using the checklist.

**Discuss Self-Care Practice**  **10 minutes**

- What emotions might you feel? The following are some examples:
  - Feelings of shame, guilt, self-doubt, embarrassment, difficulty sleeping, reduced job satisfaction, anxiety about future errors, increased burnout
- How to care for yourself when you make a mistake? The following are some examples:
  - Talking about the error is central to recovery, reach out to your program director, accepting responsibility, maintain self-care (diet, exercise, sleep)
- What should we do when a colleague makes a mistake and how can we make it feel safe to talk about mistakes? The following are some examples:
  - It's ok to be vulnerable, be authentic, allow others see you process “failure” and “mistakes”
    - How did you feel when your preceptors shared these moments with you?
      - Personal stories recounted by preceptors encouraged students and helped them understand that physicians are human and that errors will occur.
- Review the four-stage therapeutic approach for self-forgiveness described by Cornish et. Al *(Reference below).*
  - **Responsibility:** To move toward genuine self-forgiveness, the offending person takes responsibility for one’s actions and the effects of those actions. Blame shifting is minimized.
  - **Remorse:** As a result of responsibility, the offending person may experience a wide range of emotions. Shame-based (i.e., global) responses should be worked through and reduced, leaving behind more appropriate remorse-based (i.e., offense specific) responses such as guilt and regret. *(Remind students of the difference between shame and guilt. Brene Brown shares. “Shame is a focus on self, guilt is a focus on behavior. Shame is ‘I am bad.’ Guilt is ‘I did something bad.’”)*
  - **Restoration:** is an action-oriented step that follows from responsibility and remorse. In genuine self-forgiveness, the offending person seeks to make amends and repair that which was damaged to the extent possible. Behavior patterns that led to the offense are also addressed and the values violated by the offense are reaffirmed.
  - **Renewal:** Finally, in renewal, the offending person obtains the emotional state of self-forgiveness, involving renewed compassion, acceptance, and respect for oneself. Moral growth has occurred from the process of working toward self-forgiveness.

**Final thoughts/Wrap up:**  **5 minutes**

- Ask the students to consider what steps to take as an intern to familiarize themselves with the Hosptial’s error reporting process and system. The following are some examples:
  - Familiarize yourself with your Institutions Error reporting system and Patient Safety/Quality Improvement and Risk Management departments
  - At Northwell, you can report information anonymously to **Improve North**
    - Improve North
      - Where incidents should be reported
      - An enterprise-wide patient safety reporting system that enhances Northwell’s commitment to quality care and a culture of safety
      - Designed to capture adverse events/occurrences, near misses, good catches, and patient feedback including compliments, complaints and grievances
      - Provides administrative tools to assist in achieving Northwell’s goals of eliminating preventable harm and providing the safest healthcare
      - Allows for the provision of real time and scheduled alerts to enhance the timeliness of patient safety reporting
      - Provides standardized enterprise reporting at the site and system level (Safety and quality dashboards allow for sharing information across the organization)

**Take Home Points:**

- Disclosing an error is a professional obligation
- Appreciate the emotional impact of errors on patients and care providers

Following debrief, invite students to complete Survey *(Appendix G)*

**References:**

1. https://fs.blog/brene-brown-guilt-shame/#:~:text=Shame%20is%20a%20focus%20on,%2C%20%E2%80%9CI'm%20sorry.
2. Dossett L, Miller J, Jagsi R, Sales A, Fetters MD, Boothman RC, Dimick JB. A Modified Communication and Optimal Resolution Program for Intersystem Medical Error Discovery: Protocol for an Implementation Study. JMIR Res Protoc. 2019 Jul 2;8(7):e13396. doi: 10.2196/13396. PMID: 31267984; PMCID: PMC6632107.
3. Cornish, M. A., & Wade, N. G. (2015). A therapeutic model of self‐forgiveness with intervention strategies for counselors. *Journal of Counseling & Development*, *93*(1), 96-104.
