# Supplementary material for: Medical Error Disclosure: An Entrustable Professional Activity During an Objective Standardized Clinical Examination for Clerkship Students
Source: MedEdPORTAL. 2024 Feb 20;20:11382. doi: 10.15766/mep_2374-8265.11382 (PMC10876916; doi:10.15766/mep_2374-8265.11382)
Supplement: Supplementary file 1 — Faculty OSCE Guide.docxError Disclosure Standardized Patient Case.docxFaculty OSCE Checklist.docxCase-Based Experience Faculty Guide.docxCase-Based Experience Debrief Case.docxCase-Based Experience Observer Checklist.docxStudent Survey.docx [file mep_2374-8265.11382-s001.zip › _Educational Summary Report_11382.pdf]

MedEdPORTAL® The AAMC Journal of Teaching and Learning Resources

Original Publication · Open Access

# Medical Error Disclosure: An Entrustable Professional Activity During an Objective Standardized Clinical Examination for Clerkship Students

Rebecca Dougherty, MD, MSEd*, Alice Fornari, EdD, Gino Farina, MD, Doreen M. Olvet, PhD

*Corresponding author: rebecca.h.dougherty@hofstra.edu

## Abstract

**Introduction:** Most health care providers will be involved in a medical error during their careers. It is critical that future physicians receive formal training on error disclosure. **Methods:** We designed a formative skills-based objective standardized clinical exam (OSCE) for fourth-year medical students to assess competence in disclosing an error during a required entrustable professional activity. Faculty observed the encounter and completed a checklist evaluating students' performance in communication skills and content knowledge. Students received immediate formative feedback. They then participated in a facilitated case-based experience, discussed the critical elements of disclosure, utilized role-play to reinforce skills, and reflected on self-care practices. Finally, students completed a survey evaluating their perception of the OSCE's impact on their disclosure knowledge, skills, and attitudes. **Results:** Ninety-two students participated in the OSCE. Of those, 67 (73%) completed a retrospective pre/post survey assessing their disclosure knowledge, skills, and attitudes. Forty-one (62%) did not identify the error. Students who identified the error (26, 39%) were more likely to use the two-patient identifier than students who did not identify the error, $\chi^2(1) = 13.3$, $p < .001$. Self-reported comfort and confidence in disclosure improved, as did self-care practices ($ps \leq .005$). **Discussion:** Students agreed that health care providers should disclose an error and know how to do so. Student self-reported comfort in disclosure and knowledge of how to disclose and how to report an error all improved following the OSCE and structured debrief. The OSCE and case-based experience can be adapted for implementation in curricula about error disclosure.

**Keywords**
EPA, Error Disclosure, Case-Based Learning, Clinical Skills Assessment/OSCEs, Competency-Based Medical Education (Competencies, Milestones, EPAs), Professionalism, Quality Improvement/Patient Safety, Standardized Patient

## Educational Objectives

By the end of this activity, learners will be able to:

1. Describe the components of error disclosure.
2. Disclose a medical error to a standardized patient utilizing disclosure communication skills.
3. Describe comfort and confidence in error disclosure communication skills.
4. Identify factors that contribute to errors.
5. Describe how to perform self-care practices following an error and how to care for a colleague following an error.

**Citation:**
Dougherty R, Fornari A, Farina G, Olvet DM. Medical error disclosure: an entrustable professional activity during an objective standardized clinical examination for clerkship students. *MedEdPORTAL*. 2024;20:11382. https://doi.org/10.15766/mep_2374-8265.11382

## Introduction

To provide equitable care for all, providers must adhere to the quintuple aim,[1] which requires a commitment to the following: health equity, clinician well-being, pursuit of better health, improved outcomes, and lower costs. These aims build off of the landmark report *To Err Is Human,*[2] released more than 20 years ago. This report highlighted the number of preventable deaths as a result of medical error. In a study released in 2016, researchers found that medical error is the third leading cause of death.[3] Importantly, the Institute of Medicine concluded that most errors occur are not a result of incompetent clinicians but are due to inadequate systems and processes that fail in error prevention. A subsequent report, *Crossing the Quality Chasm,*[4] provided a directive to improve the quality of care being delivered such that all care would be safe, timely, effective, equitable, efficient, and patient centered.

To address these aims, the AAMC has put forth the following recommendations in its recently published *Quality Improvement*

Copyright © 2024 Dougherty et al. This is an open-access publication distributed under the terms of the Creative Commons Attribution license.

*and Patient Safety Competencies Across the Learning Continuum*[5]: Physicians must be able to demonstrate knowledge of how to disclose an error, disclose an error, and role model the disclosure of a patient safety event.[5] Patient safety and quality improvement constitute a core competency in the AAMC's core entrustable professional activities (EPAs), as EPA 13 assesses learners' ability to "identify system failures and contribute to a culture of safety."[6] Though most physicians agree that error disclosure is essential, many lack formalized training in the subject.[7,8] Only a third of physicians report formalized training in disclosure despite it being critically important.[9] Physicians also acknowledge that their initial experience with disclosure is with patients, not in a role-play exercise and not with standardized patients (SPs).[9] Identified areas for innovation include providing real-time formative feedback to learners during simulation exercises and repeat testing at a later time utilizing objective standardized clinical exams (OSCEs) to assess retention.[9] A recent scoping review on studies of disclosure found that only five studies utilized a structured assessment and did not assess skills retention.[9] Our curriculum addresses gaps in the literature identified in the scoping review of the literature on teaching medical error disclosure,[9] providing real-time formative feedback from a faculty observer and SP, as well as an additional opportunity to repractice disclosure and receive peer feedback, thereby expanding on existing disclosure curricula.[10] Finally, we include an opportunity to discuss self-care postdisclosure as this is critically important in developing strategies to support clinician resilience.

Error disclosure is a communication exchange grounded in trust and is inclusive of an acknowledgment of the occurrence of an error by the health care provider to the patient and/or the patient's family or health care proxy that also describes what occurred, what is being done to address the error, and what processes will be put in place to prevent future occurrences.[11] To assure that physicians are prepared to meet these goals, introduction to the requisite knowledge, skills, and attitudes about disclosure should occur early in the medical education continuum. The deliberate integration of curricula that address disclosure helps to create a culture built on patient centeredness, reinforces trust between patient and provider, and highlights that error disclosure is a professional obligation. The goal of our session is to provide an opportunity for the fourth-year medical students to practice and learn the skill of error disclosure using a generalizable approach. It provides a foundation on which learners can build further expertise during residency and in continuing professional development.

This project is both relevant and integral to the mission of AAMC, which has endorsed the importance of addressing issues connected to patient safety and quality improvement, including error disclosure. The project is generalizable to other institutions, medical schools, and graduate medical education and as continuing professional development for physicians.

## Methods

### Setting

The Donald and Barbara Zucker School of Medicine (ZSOM) partnered with the Clinical Skills Center (CSC) at the Northwell Health Center for Learning and Innovations (CLI) for the administration of clinical skills assessments. The CSC at CLI consisted of 14 rooms designed to resemble an inpatient examination room. ZSOM students completed OSCEs starting in their first year of medical school and were familiar with CLI, OSCE exams, and SPs.

The Hofstra University Institutional Review Board deemed this project exempt from ethical review.

### Curricular Context

We designed a disclosure curriculum as a formative assessment of student's knowledge, skill, and attitudes about error disclosure utilizing cognitive constructivism, experiential and transformative learning theories, and Kolb's self-regulated learning a framework.[12] Cognitive constructivism was employed as students were encouraged to critically reflect on their experiences with disclosure both in the clinical setting and during the OSCE. We utilized experiential and transformative learning as the students experienced error in a simulation in a standardized scenario and then were asked to reassess their approach and reflect. The curriculum allowed for formative feedback as well as a repeat exercise to assess skills retention and reflection on the experience during a debrief. The curriculum built on the training students had received on sharing emotionally challenging news during their preclerkship communications course.

The disclosure curriculum was conducted during the fourth year of medical school and was implemented starting in the summer of 2022 during students' required multistation EPA session. There were four components to the multistation EPA: a simulation session, a skills-based session focusing on IV/phlebotomy, a library session, and an OSCE. Groups of around six students completed the OSCE at one time with equal numbers of SPs and faculty observers. Students received a 15-minute general orientation and were instructed by a faculty member that they would be obtaining informed consent from a patient for a blood transfusion, starting an IV, and drawing blood. IV, phlebotomy,

Copyright © 2024 Dougherty et al. This is an open-access publication distributed under the terms of the Creative Commons Attribution license. 

and patient safety practices were reviewed. Following orientation, students had the opportunity to practice the skills of IV and phlebotomy prior to the OSCE. Next, students completed a 35-minute OSCE and 45-minute case-based learning experience. Several formative assessment tools were utilized, including repractice of the disclosure, real-time feedback from the SP and observing faculty, role-play with peers, and faculty and peer checklists. Students were familiar with and had had significant exposure to learner-centered peer feedback throughout the 4-year ZSOM curriculum, and this model continued to be used during the case-based learning experience.

Faculty participated in a 35-minute faculty development, which featured a review of the faculty OSCE guide (Appendix A), the error disclosure SP case (Appendix B), and the faculty OSCE checklist inclusive of an assessment to evaluate students' performance in communication and content knowledge (Appendix C). The assessment checklist included eight yes/no questions and one entrustment rating scale question. Faculty facilitating the case-based experience received development, including a review of the case-based experience faculty guide (Appendix D), the case-based experience case (Appendix E), the observer checklist (Appendix F), and the approach to self-care principles outlined by Cornish and Wade in "A Therapeutic Model of Self-Forgiveness With Intervention Strategies for Counselors."[13] Faculty facilitating the case-based experience led a discussion.

SPs were recruited by the CSC from its regular pool of previously screened and trained SPs. SPs received 3 hours of training for this session. SPs also received 3 hours of training focused specifically on feedback. They were trained to facilitate the learner's self-reflection with an open-ended question, "How did that go for you?", and to respond with curiosity and empathy. SPs were instructed to provide the patient perspective, noting what the learner did effectively, what the learner might like to do differently, and how the disclosure made them feel as a patient. The only criterion for SP recruitment was the ability to portray the patient. As per CLI standard protocol, SPs were trained to the role and in providing feedback to students.

The learning objectives were connected to the educational strategy and assessment methods. For the first learning objective (describe the components of error disclosure), an interactive discussion during the case-based experience (Appendix D) served as the educational strategy and was assessed by the faculty OSCE checklist (Appendix C) and peer assessment using the case-based experience observer checklist (Appendix F). For the second learning objective (disclose a medical error to an SP utilizing disclosure communication skills), the error disclosure OSCE (Appendix B) and small-group case-based experience (Appendix D) served as the educational strategy and were assessed by the faculty OSCE checklist (Appendix C) and peer assessment using the case-based experience observer checklist (Appendix F). For the third learning objective (describe comfort and confidence in error disclosure communication skills), interactive discussion and reflection during the small-group case-based exercise (Appendix D) served as the educational strategy and were assessed by the student pre/post survey (Appendix G). For the fourth learning objective (identify factors that contribute to errors), interactive discussion, reflection during the small-group case-based exercise (Appendix D), and participation in the small-group faculty-facilitated discussion served as the educational strategy and were assessed by the student pre/post survey (Appendix G). For the fifth learning objective (describe how to perform self-care practices following an error and how to care for a colleague following an error), interactive discussion and reflection during the case-based experience (Appendix D) served as the educational strategy and were assessed by participation in discussion during the case-based experience.

### Curricular Design

*OSCE development and implementation:* The design of the OSCE and case-based experience small-group session was grounded in the AAMC's *Quality Improvement and Patient Safety Competencies Across the Learning Continuum,*[5] which recommended that physicians must be able to demonstrate knowledge of how to disclose an error, disclose an error, and role model the disclosure of a patient safety event,[5] and in the AAMC's EPA 13, which assessed learners' ability to "identify system failures and contribute to a culture of safety."[6]

The 35-minute OSCE included two parts: 20 minutes for the OSCE case and 15 minutes for real-time feedback. First, the student received a verbal handoff from the faculty member. The student saw Michael or Maria Miller, a man or woman around 50 years of age with symptomatic severe anemia secondary to a gastrointestinal bleed due to nonsteroidal anti-inflammatory drug use. The student was told by the faculty member during sign-out to obtain informed consent for the blood transfusion, draw a type and cross on the task trainer, and replace the patient's IV, which had infiltrated. During the encounter, the faculty member was in the room in the role of the nurse to hand the patient the tubes and labels for the blood draw and tell the student that the IV had infiltrated. A medical error was built into the case. The patient identifiers documented on the SP's wristband and task trainer differed from those on the labels for the type and cross. The faculty member as nurse handed the student an incorrect

Copyright © 2024 Dougherty et al. This is an open-access publication distributed under the terms of the Creative Commons Attribution license.

label. The student had to identify the error by cross-checking the label with the patient's wristband prior to drawing the blood. If the student did not identify the error, the faculty member playing the nurse did not point it out. If the student did identify the error, the faculty member playing the nurse acknowledged that the student was correct, briefly left the room, returned with new correct labels, and said, "My mistake, I must have picked up the wrong label."

Upon conclusion of the initial encounter, the faculty and student exited the room. Outside the room, the faculty (still playing the role of nurse) shared the error with the student and told the student that they needed to share the error with the patient and redraw blood. This information was shared with the student even if the student had correctly identified the error. If the student had identified the error, the faculty acknowledged this but asked the student to practice the skill of error disclosure with the patient. Next, the student received real-time formative feedback from the observing faculty and SP and had the opportunity to repractice the disclosure.

The content of the case was developed by a senior emergency medicine clinician educator and was intended to replicate an authentic clinical experience that clinicians were likely to experience. The error replicated in the case often caused a delay in care and required repeating the procedure. For background, at our institution, nurses often completed blood draws. There was a policy in place that all tubes of blood and specimens be labeled at the bedside after checking two patient identifiers—name and date of birth—yet this mistake still happened. When orders were written for blood work, a label maker would print up the labels. The label makers were scattered throughout the patient care areas, and often there might be several labels waiting to be collected. In haste, it was easy to pick up the wrong label, especially when two names were similar, as in the example we used. In our scenario, the nurse, in an attempt to be helpful, picked up the wrong labels and placed them by the tube of blood and the phlebotomy/IV setup that they had prepared to be used on the patient. It was the responsibility of the person drawing the blood to use two patient identifiers at the bedside before labeling the tube of blood. The students had been told this before the exercise, during an IV/phlebotomy practice session.

*Faculty guide and checklist:* We developed the faculty OSCE guide (Appendix A) to assist in the delivery of the session. The faculty OSCE checklist (Appendix C) was developed using language from EPA 13 and the Agency for Healthcare Research and Quality (AHRQ) Communication and Optimal Resolution Toolkit, which provided instructions on how to complete an error disclosure[14] and an entrustability scale that assessed the student's entrustment on error disclosure. If the student could disclose the error utilizing the skills outlined, faculty indicated that the student could be entrusted in that component.

During the encounter, faculty observed the students and completed the checklist in real time. If a student identified the error, the faculty acknowledged this and shared with the student that they were expected to perform the error disclosure with the SP. During the faculty-facilitated debrief with the SP, students were encouraged to reflect on their perceptions of what they had done effectively and what they hoped to improve and also received formative feedback. SPs provided reflections on their experience as the patient regarding how the error disclosure made them feel. Students had the opportunity to repractice their disclosure communication skills with the SP.

*Case-based learning exercise:* Following the OSCE, students participated in a case-based learning exercise facilitated by a faculty member using the facilitator guide (Appendix D), with an average of six students per group. Students reflected on whether or not they had personally participated in or observed error disclosure during their clinical experiences and, if so, what they had observed and how it made them feel. They also reflected on the importance of disclosure and discussed the critical elements of disclosure, utilizing rolling role-play to repractice.

We designed the role-play case and observer checklist to allow students to work in triads, providing each student the opportunity to play the role of the clinician, patient, and observer when utilizing the provided case (Appendix E). Student observers received a checklist (Appendix F) to assess their peers' performance. The checklist included the key error disclosure elements adapted from the AHRQ error disclosure communication skills checklist[14] on which students were assessed by faculty. Our students were familiar with role-play and the provision of learner-centered peer feedback as this model was used for our communication curricular sessions. The students gave each other formative feedback utilizing the checklist. Finally, the faculty guided a discussion on self-care principles utilizing a therapeutic model of self-forgiveness,[13] which included four steps: to accept responsibility for one's actions, to engage in remorse-based emotional response, to commit to action-oriented restoration, and finally, renewal. Students were asked to consider how they would use this framework to care for themselves and/or a colleague following error disclosure.

Copyright © 2024 Dougherty et al. This is an open-access publication distributed under the terms of the Creative Commons Attribution license. 

*Student survey:* At the completion of the case-based experience, all students were asked to fill out a retrospective pre/post survey (Appendix G) that assessed several elements, including their attitudes about error disclosure; reflections on their experience in the OSCE; whether they had identified the error; if so, what factors contributed to the recognition; and if not, why not. The students completed the survey anonymously. The retrospective pre/post survey design was a method of assessing self-reported changes in knowledge, skills, and attitudes as the result of an intervention by having respondents rate their level after the intervention and retrospectively assess preintervention levels.[15,16] This was thought to minimize response shift bias, which can occur when respondents complete a preintervention survey using a different frame of reference than when they complete a postintervention survey.[17,18] At the preintervention time point, learners may not accurately assess their baseline knowledge, skills, and attitudes because they don't know what they don't know. The retrospective pre/post design was also less burdensome for respondents to complete and reduced the likelihood of missing data.[19]

Students were provided with a QR code to scan to access the survey, administered in Qualtrics. The survey assessed students' perception of disclosing errors using five statements that they were asked to rate using a 5-point Likert scale (1 = *strongly disagree,* 2 = *disagree,* 3 = *neither agree nor disagree,* 4 = *agree,* 5 = *strongly agree*). Several open-ended questions also asked students to reflect on how they felt about the encounter and what contributed to their either identifying or not identifying the error.

### Statistical Analysis

We conducted a mixed-methods study, analyzing qualitative and quantitative data. Quantitative data were statistically evaluated using IBM SPSS Statistics Version 28.0 (SPSS, Inc.). Descriptive statistics were presented as the number and percentage of student respondents who strongly agreed/agreed with each of the seven individual items on the 5-point Likert survey. For the faculty checklist, the number and percentage of yes responses were presented for each of the individual items, as well as the distribution of responses to the 5-point entrustment scale. A Wilcoxon signed rank test was used to compare students' pre/post survey responses. A chi square test was used to compare between groups (students who identified the error vs. students who did not) on binary faculty checklist items, and the Mann-Whitney $U$ test was used for the ordinal entrustment score. For all tests, $p \leq .05$ was considered statistically significant.

Qualitative data from open-ended survey questions were analyzed using inductive content analysis[20] to obtain themes from the students' narrative responses. One coder (Rebecca Dougherty) initially read through the qualitative responses to each question, coding phrases and identifying categories to organize the codes into meaningful units. A second coder (Doreen M. Olvet) reviewed the coded data, noting when there was a disagreement with the original categorization. The two coders met to discuss any discrepancies in coding until a consensus was reached.

### Personnel and Materials

The disclosure curriculum required an SP, faculty, and task trainer arm IV materials.

## Results

### Student Checklist Data

Ninety-two students participated in the OSCE. Of those, 67 students (73%) completed a retrospective pre/post survey that assessed their disclosure knowledge, skills, and attitudes. Survey data for both pre- and postsession were matched for 51 students. The remaining 16 students had been given a previous version of the survey asking only for the postsession data; therefore, they were not included in the pre/post survey analysis. Figure 1 shows the number and percentage of students at each survey stage, including 41 students (62%) who did not identify the error and 26 (39%) who did.

### Faculty Checklist Data

Of the 67 students who completed the retrospective pre/post survey, there was faculty checklist data for 58 (87%) as not all faculty either completed the checklist or submitted it. The Table shows the number and percentage of students who correctly performed each task on the faculty checklist ($N = 58$). Faculty checklists were available for 36 students (62%) who did not identify the error. Students who identified the error were more likely to use the two-patient identifier when labeling the blood

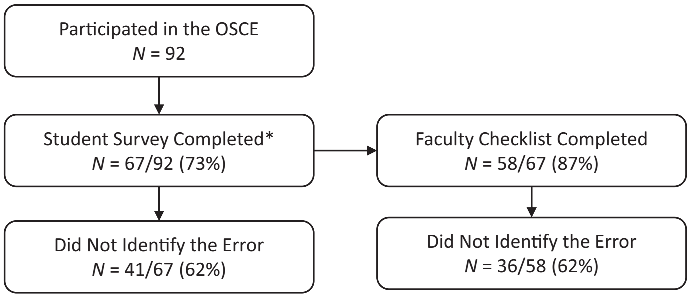


**Figure 1.** Flowchart depicting the sample of students having data available for the student survey and faculty checklist. *Sixteen students had only postsurvey data.

Copyright © 2024 Dougherty et al. This is an open-access publication distributed under the terms of the Creative Commons Attribution license.

**Table.** Number (%) of Students Correctly Performing Each Task on the Faculty Checklist Based on Whether They Identified the Error ($N = 22/58$, 37%) or Did Not ($N = 36/58$, 62%)

| | No. (%) | |
|---|---|---|
| **Item** | **Identified the Error** | **Did Not Identify the Error** |
| Did the student: | | |
| Use the two-patient identifier when labeling the blood tube?[a] | 14 (64) | 6 (17) |
| Explore the patient's understanding of what happened? | 19 (86) | 30 (83) |
| Disclose the error using clear language? | 19 (86) | 30 (83) |
| Discuss steps being taken to manage the event? | 22 (100) | 35 (97) |
| Express regret/offer an apology? | 22 (100) | 35 (97) |
| Discuss future steps to avoid a similar event? | 20 (91) | 32 (89) |
| Utilize the micro-skills of empathy during the encounter? | 22 (100) | 36 (100) |
| Reflect on the underlying factors which contribute to errors?[b] | 18 (100) | 28 (86) |
| Identify and reflect on their own contribution to the error?[b] | 18 (100) | 27 (96) |
| Know how to seek help following an error?[b] | 17 (94) | 26 (93) |
| EPA-13 Entrustment score | | |
| Can only observe someone doing it | 0 (0) | 0 (0) |
| Can do it if supervisor talks them through it | 0 (0) | 0 (0) |
| Can do it with supervisor in the room to direct them from time to time | 1 (5) | 1 (3) |
| Can do it with supervisor available just in case | 4 (18) | 15 (42) |
| Can do it without supervisor in the room | 17 (77) | 20 (56) |

Abbreviation: EPA, entrustable professional activity.
[a] $p < .001$
[b] $N = 46$.

tube than students who did not identify the error, $\chi^2(1) = 13.3$, $p < .001$. There was no significant difference between the two groups on any other checklist item (all $ps > .05$). For the entrustment score, 77% of the students who identified the error and 56% of the students who did not achieved the highest rating, but the difference between the two groups was not statistically significant ($U = 315.5$, $p = .13$).

### Retrospective Pre/Post Student Survey Data

We evaluated students' knowledge, skills, and attitudes in a retrospective pre/post survey that they completed following the case-based experience. Figure 2 shows the percentage of students who strongly agreed/agreed with each survey item before and after the survey ($N = 51$). There was a statistically significant increase in agreement for all seven items (all $ps \leq .005$). Self-reported comfort and confidence in disclosure improved as did confidence in knowledge of error reporting and self-care. There was no significant difference in agreement when comparing students who identified the error and those who did not (all $ps > .05$).

### Qualitative Student Survey Data

Figure 3 shows the categories that were identified through a content analysis of the open-ended comments from the student survey. Of those who did identify the error ($N = 18$), students reported feeling comfortable speaking up about it. Students responded to the prompt "What do you think contributed to your identifying the error?" by citing double-checking and following steps in a protocol as reasons: "Reminding myself what others do, which is to double check everything." Students who identified the error felt "good" and "comfortable" disclosing it to the patient.

Thirty-three students who did not identify the error reported feeling emotions such as embarrassment, guilt, disappointment, and shame: "I felt shame for not noticing the name discrepancy; I felt embarrassed that I didn't catch something that I felt should have been obvious and began judging myself for it." Students responded to the prompt "What did you think contributed to you not identifying the error?" by sharing explanations such as a lack of familiarity with the procedure, being preoccupied, assuming things would be correct, and rushing: "Being in a new situation doing a procedure that was new to me; I was too focused on making sure that the patient, who was afraid of needles, was comfortable." Of the students who did not identify the error, some reported feeling "comfortable" and "relieved" disclosing the error to the patient, whereas others felt "awkward," "ashamed," "uncomfortable," and/or "guilty."

Twenty-four students (47%) had observed an error disclosure during their clinical rotations. Most students indicated that the errors had been properly disclosed to the patient—"The error disclosure was disclosed in a positive way and handled very responsibly"—and that "the patient was frustrated but appreciated the apology and explanation given." However, there were some comments indicating that error disclosure was poorly handled—"the attending responsible never talked with the patient and relied on others"—or was "done in a halfhearted way."

Copyright © 2024 Dougherty et al. This is an open-access publication distributed under the terms of the Creative Commons Attribution license.

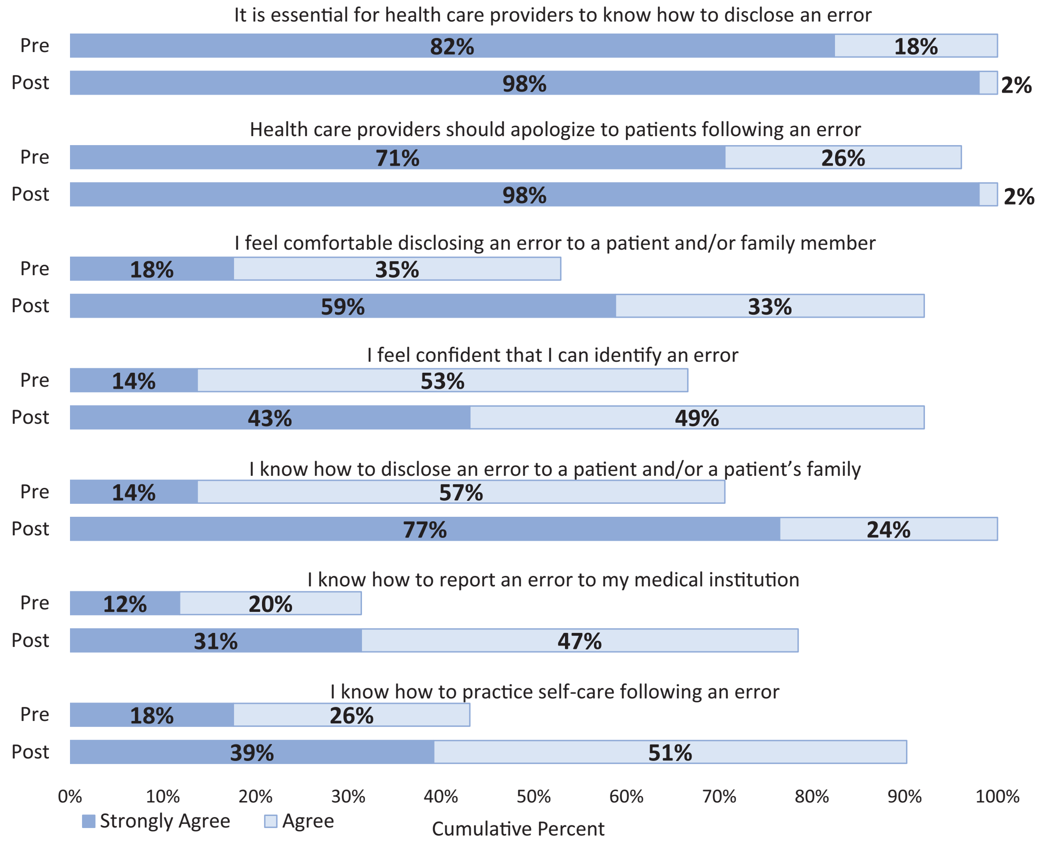


**Figure 2.** Percentage of students who strongly agreed/agreed with each survey item before and after the survey ($N = 51$). There was a statistically significant increase in agreement for all seven items (all $ps \leq .005$).

| **Identified the Error (*N* = 18)** | | | **Did Not Identify the Error (*N* = 33)** | | |
|---|---|---|---|---|---|
| Briefly describe how you felt about speaking up about the error in the moment. | Comfortable | 11 (61%) | Briefly describe how you felt after realizing that you didn't identify the error. | Guilty/ashamed | 17 (52%) |
| | Awkward/difficult | 4 (22%) | | Disappointed (in self) | 11 (33%) |
| | Confused | 3 (17%) | | Nervous | 4 (12%) |
| What do you think contributed to your identifying the error? | Double-checking | 10 (56%) | What do you think contributed to your NOT identifying the error? | Lack of familiarity with procedure | 11 (33%) |
| | Following steps in the protocol | 4 (22%) | | Assuming it would be correct | 8 (24%) |
| | Primed for an error | 2 (11%) | | Preoccupied with task | 5 (15%) |
| | | | | Rushing | 5 (15%) |
| | | | | Trusting the process | 3 (9%) |
| Please reflect on the experience of disclosing the error to the patient. How did you feel? | Good | 7 (39%) | Please reflect on the experience of disclosing the error to the patient. How did you feel? | Good | 11 (33%) |
| | Comfortable | 4 (22%) | | Comfortable | 5 (15%) |
| | Awkward/difficult | 4 (22%) | | Awkward/difficult | 6 (18%) |
| | Guilty | 3 (17%) | | Guilty/ashamed | 4 (12%) |
| | | | | Uncomfortable | 6 (18%) |

**Figure 3.** Categories derived from the content analysis of the open-ended questions on the retrospective pre/post student survey based on whether students identified the error ($N = 18$) or did not ($N = 33$).

Copyright © 2024 Dougherty et al. This is an open-access publication distributed under the terms of the Creative Commons Attribution license.

## Discussion

It is critical that physicians receive training in error disclosure as it is likely they will be involved in a medical error sometime during their professional career. Students receive foundational knowledge and skills on error disclosure from this curriculum upon which they can build further expertise. An identified area for curricular innovation based on literature review was to provide opportunities for trainees to have their initial disclosure experience in a role-play exercise and/or with SPs.[9] Additional opportunities included providing formative feedback to learners during simulation exercises, a structured assessment, and repeat testing at a later time to assess skills retention.[9] Strengths of this curriculum are that it promotes the development of skills in error disclosure during simulation, provides formative feedback to learners on their demonstrated skills, and offers an opportunity to repractice, as well as a structured assessment, all filling identified curricular gaps. The OSCE and case-based experience can be adapted for implementation in curricula about error disclosure. The error disclosure checklist can be applied to any error scenario in which the goal is to assess disclosure.

We learned that students agreed health care providers should disclose an error and should have the knowledge and skill to do so. Students' comfort in disclosure and knowledge of how to disclose and how to report an error all improved following the OSCE and structured debrief. Confidence in identifying an error increased following the OSCE, as did self-care. The outcomes show that students valued the opportunity to practice the skill of error disclosure as many had not witnessed an error disclosure during their clinical rotations. Students also reported experiencing shame and embarrassment associated with error disclosure, which are emotions that can impact resilience. Students received training in self-care and were appreciative of the exercise as it allowed time for them to reflect on their clinical experiences through the lens of error disclosure.

An additional strength of this curriculum is that it encourages reflection on self-care following error disclosure. This builds off of the Sharing Emotionally Challenging News curriculum to which our students are exposed in their preclinical year. The curriculum uses transformative learning theory, having the students experience error disclosure and then receive formal didactic exposure to the critical components of disclosure. The curriculum also allows students to self-assess their utilization of those key steps. In the quantitative analysis, some students reported feelings of guilt and/or embarrassment, emotions that are described in the literature around second victims.[21] Students were provided with the opportunity to share these emotions during the case-based small-group experience, which focused on self-care following an error. The curriculum achieved our goals and objectives as well as identifying strengths, limitations, and areas of focus for future directions.

Limitations of the project include self-reported change and resources such as faculty time, SP training and time, and materials including task trainers and phlebotomy kits. Students who successfully identified the error were also asked to practice the skill of error disclosure, which may have caused an awkward experience for some. The goal, however, was to practice the skill of disclosure, and as the students were well versed in role-play, a teaching strategy utilized throughout the 4-year curriculum, they were asked to complete the disclosure irrespective of whether or not they had identified the error. This may have created a different experience for those who identified the error versus those who did not and will be explored as a free-text item in the student survey moving forward.

Future directions include providing enhanced faculty development around the faculty OSCE checklist assessment that will include interrater reliability testing, repeating a similar OSCE at a later time during the fourth year of medical school to further assess for spaced skill retention, and assessing students' perception of the impact of formative feedback from the SP. We plan further curricular development around the assessment of self-care practices following error disclosure, including adding care of a colleague following an error as an additional OSCE. Participating faculty facilitators will be encouraged to become error disclosure champions in their clinical environments, to reinforce opportunities for exposure to these conversations in the clinical environment, and to allow further authentic training for students on the skill of error disclosure. Finally, revisions to the entrustability score will be implemented, including removing "Can do it without supervisor in the room" from the faculty OSCE checklist entrustment scale and adding a qualitative piece for faculty to comment on student performance so as to describe students' strengths and opportunities for improvement.

## Appendices

A. Faculty OSCE Guide.docx
B. Error Disclosure Standardized Patient Case.docx
C. Faculty OSCE Checklist.docx
D. Case-Based Experience Faculty Guide.docx
E. Case-Based Experience Debrief Case.docx

Copyright © 2024 Dougherty et al. This is an open-access publication distributed under the terms of the Creative Commons Attribution license.

F. Case-Based Experience Observer Checklist.docx

G. Student Survey.docx

*All appendices are peer reviewed as integral parts of the Original Publication.*

**Rebecca Dougherty, MD, MSEd:** Associate Professor, Department of Medicine and Science Education, Zucker School of Medicine at Hofstra/Northwell; ORCID: https://orcid.org/0000-0002-7177-982X

**Alice Fornari, EdD:** Professor, Department of Family Medicine and Science Education, Zucker School of Medicine at Hofstra/Northwell; ORCID: https://orcid.org/0000-0001-5475-2732

**Gino Farina, MD:** Professor, Department of Emergency Medicine and Science Education, Zucker School of Medicine at Hofstra/Northwell

**Doreen M. Olvet, PhD:** Associate Professor, Department of Science Education, Zucker School of Medicine at Hofstra/Northwell

**Acknowledgments**
Terry Wanamaker, MSEd, Standardized Patient Educator, Northwell Center for Learning and Innovation, was instrumental to this initiative.

**Disclosures**
None to report.

**Funding/Support**
None to report.

**Ethical Approval**
The Hofstra University Institutional Review Board deemed further review of this project not necessary.

## References

1. Itchhaporia D. The evolution of the quintuple aim: health equity, health outcomes, and the economy. *J Am Coll Cardiol*. 2021;78(22):2262-2264. https://doi.org/10.1016/j.jacc.2021.10.018
2. Institute of Medicine. *To Err Is Human: Building a Safer Health System*. National Academies Press; 2000. https://doi.org/10.17226/9728
3. Makary MA, Daniel M. Medical error—the third leading cause of death in the US. *BMJ*. 2016;353:i2139. https://doi.org/10.1136/bmj.i2139
4. Institute of Medicine. *Crossing the Quality Chasm: A New Health System for the 21st Century*. National Academies Press; 2001. https://doi.org/10.17226/10027
5. Association of American Medical Colleges. *Quality Improvement and Patient Safety Competencies Across the Learning Continuum*. Association of American Medical Colleges; 2019.
6. Amiel JM, Andriole DA, Biskobing DM, et al. Revisiting the core entrustable professional activities for entering residency. *Acad Med*. 2021;96(7S):S14-S21. https://doi.org/10.1097/ACM.0000000000004088
7. White AA, Gallagher TH, Krauss MJ, et al. The attitudes and experiences of trainees regarding disclosing medical errors to patients. *Acad Med*. 2008;83(3):250-256. https://doi.org/10.1097/ACM.0b013e3181636e96
8. Gardner AK, Lim G, Minard CG, Guffey D, Pillow MT. A cross-specialty examination of resident error disclosure and communication skills using simulation. *J Grad Med Educ*. 2018;10(4):438-441. https://doi.org/10.4300/JGME-D-17-00603.1
9. Stroud L, Wong BM, Hollenberg E, Levinson W. Teaching medical error disclosure to physicians-in-training: a scoping review. *Acad Med*. 2013;88(6):884-892. https://doi.org/10.1097/ACM.0b013e31828f898f
10. Bell SK, Moorman DW, Delbanco T. Improving the patient, family, and clinician experience after harmful events: the “when things go wrong” curriculum. *Acad Med*. 2010;85(6):1010-1017. https://doi.org/10.1097/ACM.0b013e3181dbedd7
11. Fein SP, Hilborne LH, Spiritus EM, et al. The many faces of error disclosure: a common set of elements and a definition. *J Gen Intern Med*. 2007;22(6):755-761. https://doi.org/10.1007/s11606-007-0157-9
12. Kolb DA. *Experiential Learning: Experience as the Source of Learning and Development*. Prentice-Hall: 1984.
13. Cornish MA, Wade NG. A therapeutic model of self-forgiveness with intervention strategies for counselors. *J Couns Dev*. 2015;93(1):96-104. https://doi.org/10.1002/j.1556-6676.2015.00185.x
14. Dossett L, Miller J, Jagsi R, et al. A modified communication and optimal resolution program for intersystem medical error discovery: protocol for an implementation study. *JMIR Res Protoc*. 2019;8(7):e13396. https://doi.org/10.2196/13396
15. Thomas EV, Wells R, Baumann SD, et al. Comparing traditional versus retrospective pre-/post-assessment in an interdisciplinary leadership training program. *Matern Child Health J*. 2019;23(2):191-200. https://doi.org/10.1007/s10995-018-2615-x
16. Skeff KM, Stratos GA, Bergen MR. Evaluation of a medical faculty development program: a comparison of traditional pre/post and retrospective pre/post self-assessment ratings. *Eval Health Prof*. 1992;15(3):350-366. https://doi.org/10.1177/016327879201500307
17. Howard GS, Ralph KM, Gulanick NA, Maxwell SE, Nance SW, Gerber SK. Internal invalidity in pretest-posttest self-report evaluations and a re-evaluation of retrospective pretests.

Copyright © 2024 Dougherty et al. This is an open-access publication distributed under the terms of the Creative Commons Attribution license.

*Appl Psychol Meas*. 1979;3(1):1-23. https://doi.org/10.1177/014662167900300101

18. Drennan J, Hyde A. Controlling response shift bias: the use of the retrospective pre-test design in the evaluation of a master's programme. *Assess Eval High Educ*. 2008;33(6):699-709. https://doi.org/10.1080/02602930701773026
19. Raidl M, Johnson S, Gardiner K, et al. Use retrospective surveys to obtain complete data sets and measure impact in extension programs. *J Ext*. 2004;42(2):13.
20. Hsieh HF, Shannon SE. Three approaches to qualitative content analysis. *Qual Health Res*. 2005;15(9):1277-1288. https://doi.org/10.1177/1049732305276687
21. Wu AW. Medical error: the second victim. *BMJ*. 2000;320(7237):726. https://doi.org/10.1136/bmj.320.7237.726

**Received:** June 8, 2023
**Accepted:** November 14, 2023
**Published:** February 20, 2024

Copyright © 2024 Dougherty et al. This is an open-access publication distributed under the terms of the Creative Commons Attribution license.
